# Sleep Disorders in Family Medicine: A Cross-Sectional Study on Prevalence, Screening, and Treatment Approaches in Saudi Arabia

**DOI:** 10.7759/cureus.78937

**Published:** 2025-02-13

**Authors:** Marwa F AlAlawi, Najla M Alsudairy

**Affiliations:** 1 College of Medicine, Arabian Gulf University, Manama, BHR; 2 Department of Family Medicine, National Guard Hospital, Jeddah, SAU

**Keywords:** barriers, diagnostic methods, family medicine, management strategies, prevalence, primary care, saudi arabia, screening practices, sleep disorders, treatment preferences

## Abstract

Background: Sleep disorders are prevalent in the general population and can significantly impact health outcomes. Family medicine practitioners often serve as the first point of contact for patients with sleep-related issues. However, sleep disorders are frequently underdiagnosed and undertreated in primary care settings. This study investigates the prevalence, screening practices, management strategies, and barriers faced by family medicine practitioners in Saudi Arabia regarding sleep disorders.

Objective: This study aimed to assess the prevalence of sleep disorders, explore the diagnostic and treatment practices, and identify barriers to effective management among family medicine practitioners in Saudi Arabia.

Methods: A cross-sectional survey was conducted among 245 family medicine practitioners in Saudi Arabia using a structured questionnaire. The survey gathered information on demographics, sleep disorder prevalence, diagnostic methods, treatment preferences, patient knowledge, and barriers to management. Descriptive statistics were used to analyze the responses. Ethical approval was obtained, and informed consent was provided by all participants.

Results: Among the 245 participants, 22.4% reported experiencing trouble falling or staying asleep at least three times a week, and 46.2% experienced excessive daytime sleepiness. Snoring, gasping, or choking during sleep was reported by 29% of participants, with 12.9% diagnosed with sleep apnea and 23.3% diagnosed with insomnia. Regarding treatment, 41% of participants with diagnosed sleep disorders received no treatment, while pharmacological therapy (sedative medications and antidepressants) was the most common intervention (32.2%). Cognitive behavioral therapy for insomnia (CBT-I) was prescribed to 5.2% of patients, and 6.0% received continuous positive airway pressure (CPAP) therapy. Diagnostic practices revealed that 44.2% of practitioners used self-reported questionnaires like the Pittsburgh Sleep Quality Index (PSQI), while 28.1% relied only on clinical interviews. Barriers to effective management included patient awareness (32.9%), time constraints (27.8%), and limited access to mental health professionals (13.3%). A significant proportion (57.8%) of respondents believed more research was needed on sleep disorders within family medicine settings.

Conclusion: Sleep disorders are prevalent among family medicine patients in Saudi Arabia, with significant underdiagnosis and undertreatment. Pharmacological interventions are more commonly used than non-pharmacological treatments, such as CBT-I. Barriers like lack of patient awareness, time limitations, and access to mental health specialists hinder effective management. Training for family medicine practitioners on sleep disorder management and the implementation of routine screening are essential steps toward improving diagnosis and treatment in primary care settings.

## Introduction

Sleep disorders represent a significant public health concern due to their widespread prevalence and substantial impact on physical, mental, and social well-being. These disorders are commonly encountered in primary care settings, where family medicine practitioners often serve as the first point of contact for patients seeking care for a wide range of health issues, including sleep disturbances [1-3]. Conditions such as insomnia, obstructive sleep apnea, and restless legs syndrome have been linked to a variety of negative outcomes, including impaired cognitive function, reduced quality of life, and an increased risk of chronic diseases (e.g., cardiovascular disease, diabetes) and mental health conditions (e.g., depression, anxiety). Despite their high prevalence, sleep disorders often remain undiagnosed and undertreated, primarily due to insufficient screening, limited treatment options, and the complex nature of sleep-related issues [2,4].

In family medicine practices, the burden of sleep disorders is frequently underrecognized. Many patients may not seek help for their sleep issues, attributing them to stress or lifestyle factors, or may present with symptoms that overlap with comorbid conditions such as depression, obesity, or hypertension [4,5]. Family medicine practitioners are in a unique position to identify and manage sleep disorders, given their long-term relationships with patients and comprehensive knowledge of their medical history. However, the extent to which sleep disorders are adequately identified, diagnosed, and managed in these settings remains unclear.

Managing sleep disorders in primary care is multifaceted, involving both non-pharmacological and pharmacological interventions. Cognitive-behavioral therapy for insomnia (CBT-I) is often a first-line treatment, demonstrating efficacy in improving sleep quality and reducing insomnia symptoms [6,7]. However, access to trained therapists and resources for CBT-I is limited, particularly in resource-constrained healthcare settings. As a result, many family medicine practitioners rely on pharmacological treatments, such as sedative-hypnotics (e.g., benzodiazepines and zolpidem) or antidepressants, despite concerns over their long-term safety and potential for dependence [2-5].

Several barriers hinder the effective management of sleep disorders in family medicine settings. These include a lack of awareness and training in sleep medicine among family practitioners, time constraints during consultations, and the complexity of diagnosing and managing sleep disorders in patients with multiple comorbid conditions. Additionally, sleep disorders are often considered a lower priority compared to more immediate health concerns, leading to insufficient attention and resources allocated to their management [1-6].

Family medicine practitioners play a pivotal role in the identification, diagnosis, and management of sleep disorders. Due to their broad scope of practice and long-term relationships with patients, they are ideally positioned to screen for and treat common sleep-related issues [5-7]. However, studies indicate that family medicine practitioners often lack confidence and training in sleep medicine, which may contribute to missed diagnoses and suboptimal management. Furthermore, primary care providers may not routinely screen for sleep disorders, and when they do, they may lack the necessary tools or resources to make an accurate diagnosis or provide effective treatment.

This study aims to address the gap in knowledge regarding the prevalence and management of sleep disorders in family medicine practices in Saudi Arabia. By surveying family medicine practitioners about their experiences with sleep disorder screening, diagnosis, treatment, and barriers to management, the study seeks to provide valuable insights into the current state of sleep disorder care in primary care settings. The findings will inform future interventions aimed at improving the recognition and management of sleep disorders, ultimately enhancing patient outcomes and quality of life.

## Materials and methods

Study design

This cross-sectional study aimed to evaluate the prevalence and management of sleep disorders among family medicine practitioners in Saudi Arabia. A survey questionnaire was developed as the primary data collection tool, focusing on practitioners' demographic information, their experience with sleep disorders, screening methods, diagnostic tools, treatment approaches, and the barriers they encounter when managing these conditions.

Study population

The study targeted family medicine residents, fellows, and consultants practicing in various primary care settings across Saudi Arabia. Eligible participants were family medicine practitioners who are currently working in family medicine clinics. A convenience sampling method was used to select participants from multiple healthcare centers located in both urban and rural areas. Practitioners not currently engaged in family medicine or those unable to provide informed consent were excluded from the study.

Survey questionnaire

The data collection tool for this study was a structured questionnaire consisting entirely of multiple-choice questions. The questionnaire was designed to gather comprehensive information on practitioners' demographics, sleep disorder screening methods, diagnostic tools, treatment approaches, barriers to management, and their attitudes towards sleep disorder care in family medicine. It was developed in collaboration with experts in both family medicine and sleep disorders to ensure its content validity and comprehensiveness. To assess the clarity, reliability, and feasibility of the tool, the questionnaire was pre-tested on a small sample of practitioners prior to its main distribution.

The questionnaire included several sections, including demographics, sleep disorder screening, treatment for sleep disorders, management of sleep disorders, patient knowledge and awareness, barriers to managing sleep disorders and closing questions. The demographic section asked about the participants' age, gender, and years of experience in family medicine. The sleep disorders screening section inquired about the frequency of sleep difficulties, excessive daytime sleepiness, and common symptoms such as snoring, gasping, and choking during sleep. In the treatment section, the questionnaire explored the treatments prescribed for sleep disorders, including pharmacological therapies, CBT-I, and the use of continuous positive airway pressure (CPAP) machines. The management section focused on the frequency with which practitioners encounter patients with sleep disorders, the diagnostic methods they use (e.g., questionnaires, polysomnography), and their management strategies. The section on patient knowledge and awareness examined practitioners' perceptions of patients' understanding of sleep hygiene and the risks of untreated sleep disorders. The barriers section addressed issues such as limited consultation time, patient resistance to treatment, and lack of access to diagnostic tools or specialists. Finally, the closing questions explored whether practitioners believed sleep disorders were underdiagnosed and if there was a need for further research and training.

Data collection procedure

The questionnaire was distributed electronically via email and through a secure online platform to ensure easy access and completion by participants. Upon receiving the invitation to participate, respondents were informed of the study's objectives and provided with a link to the electronic consent form. Informed consent was obtained digitally, and responses were anonymized to maintain confidentiality. Ethical approval for the study was obtained from the Ethics Committee of the Ministry of Health, Saudi Arabia (25/11/1412). All data collection procedures adhered to ethical guidelines set by the research ethics committee.

Data analysis

The data were analyzed using descriptive statistics. Frequencies and percentages were calculated for categorical variables, such as gender, age groups, sleep disorder prevalence, and treatment approaches. Each section of the questionnaire was summarized to identify trends and patterns in responses. IBM SPSS Statistics software, version 26.0 (IBM Corp., Armonk, NY), was used for the analysis, and the results were presented as frequency distributions for each question.

## Results

Demographics

A total of 245 family medicine practitioners participated in the study. The majority of respondents were between the ages of 31 and 50 years, with 28.5% (70/245) in the 41-50 years age group, followed by 26.5% (65/245) in the 51-60 years range. There was a relatively balanced distribution between male (39.6%, 97/245) and female (60.4%, 148/245) participants (Table 1).

**Table 1:** Participant demographics (N=245)

Demographic factors	Response options	Frequency (n)	Percentage (%)
Age	18–30 years	12	5%
	31–40 years	45	18.5%
	41–50 years	70	28.5%
	51–60 years	65	26.5%
	61+ years	43	17.5%
Gender	Male	97	39.6%
	Female	148	60.4%

Screening of sleep disorders

Regarding the frequency of sleep-related issues, 22.4% (55/245) of respondents reported experiencing trouble falling or staying asleep at least three times a week. Excessive daytime sleepiness was reported by 46.2% (48/245) of respondents, with 53% (130/245) indicating that they never feel excessively sleepy during the day. Snoring, gasping, or choking during sleep was reported by 29% of participants (72/245), with 10.1% (25/245) experiencing it regularly. In terms of prior diagnoses, 12.9% (32/245) had been diagnosed with sleep apnea, 23.3% (58/245) with insomnia, and 51.2% (127/245) had no formal diagnosis (Table 2).

**Table 2:** Prevalence of sleep disorders and symptoms (N=245)

Question	Response options	Frequency (n)	Percentage (%)
Do you have trouble falling asleep or staying asleep at least three times a week?	Yes	55	22.4%
	No	190	77.6%
How often do you feel excessively sleepy during the day?	Never	130	53%
	Rarely (1-2 times a week)	62	25.2%
	Occasionally (3-4 times a week)	34	13.8%
	Frequently (5-7 times a week)	19	7.6%
Do you experience snoring, gasping, or choking during sleep?	Yes, regularly	25	10.1%
	Yes, occasionally	47	18.9%
	No	173	71%
Have you ever been diagnosed with a sleep disorder?	Sleep apnea	32	12.9%
	Insomnia	58	23.3%
	Restless legs syndrome	13	5.2%
	Narcolepsy	6	2.4%
	None	127	51.2%

Treatment for sleep disorders

Among those with diagnosed sleep disorders, 41.0% (102/245) were not prescribed any treatment. For those who were prescribed treatments, the most common intervention was pharmacological therapy, with 20.1% (50/245) receiving sedative medications, and 12.1% (30/245) being prescribed antidepressants. Cognitive behavioral therapy for insomnia was prescribed to 5.2% (13/245), while 6.0% (15/245) received CPAP therapy (Table 3).

**Table 3:** Prescribed treatments for sleep disorders (N=245)

Question	Response options	Frequency (n)	Percentage (%)
Which of the following treatments have you been prescribed for sleep disorders?	Sedative medications (e.g., benzodiazepines, zolpidem)	50	20.1%
	Antidepressants	30	12.1%
	Continuous positive airway pressure (CPAP) therapy	15	6.0%
	Cognitive behavioral therapy for insomnia (CBT-I)	13	5.2%
	Lifestyle modifications (e.g., sleep hygiene)	25	10.1%
	None	102	41.0%

Management of sleep disorders

In terms of sleep disorder management, 38.6% (96/245) of respondents reported frequently encountering patients with sleep disorders (four to seven cases per month), while 28.5% (71/245) reported seeing more than seven patients per month. Regarding diagnostic tools, 44.2% (110/245) of practitioners used self-reported questionnaires like the Pittsburgh Sleep Quality Index (PSQI) or the Epworth Sleepiness Scale (ESS), and 28.1% (70/245) relied solely on clinical interviews. Only 11.3% (28/245) of practitioners employed polysomnography, and 6.0% (15/245) used home sleep apnea testing (Table 4).

**Table 4:** Frequency and methods of sleep disorder assessment (N=245)

Question	Response options	Frequency (n)	Percentage (%)
How frequently do you encounter patients reporting sleep disorders?	Rarely (less than 1 per month)	17	6.8%
	Occasionally (1-3 patients per month)	61	24.5%
	Frequently (4-7 patients per month)	96	38.6%
	Very frequently (more than 7 patients per month)	71	28.5%
Which diagnostic tools do you use to screen for sleep disorders?	Self-reported questionnaires (e.g., Pittsburgh Sleep Quality Index (PSQI), Epworth Sleepiness Scale (ESS))	110	44.2%
	Polysomnography (sleep study)	28	11.3%
	Home sleep apnea testing (HSAT)	15	6.0%
	Clinical interviews alone	70	28.1%
	I do not use any diagnostic tools	20	8.0%

Treatment preferences

When asked about treatment preferences, 32.5% (81/245) of respondents preferred lifestyle modifications such as exercise and sleep hygiene as first-line treatments, followed by pharmacological therapy (16.9%, 42/245) and referral to specialists (18.1%, 45/245). Six percent (15/245) preferred cognitive behavioral therapy (CBT), while 23.3% (58/245) did not treat sleep disorders (Table 5).

**Table 5:** Preferred approaches to sleep disorder management (N=245)

Question	Response options	Frequency (n)	Percentage (%)
What is your preferred first-line treatment for sleep disorders?	Pharmacological therapy (e.g., sleep aids, antidepressants)	42	16.9%
	Cognitive behavioral therapy (CBT)	15	6.0%
	Lifestyle modifications (e.g., exercise, sleep hygiene)	81	32.5%
	Referral to a specialist (e.g., sleep physician, psychologist)	45	18.1%
	I do not treat sleep disorders	58	23.3%

Patient knowledge and awareness

Regarding patient knowledge, 38.1% (95/245) of respondents believed their patients were somewhat familiar with sleep hygiene practices, while 42.9% (107/245) felt their patients were very familiar. However, 57.8% (144/245) of respondents thought that patients with sleep disorders did not understand the risks associated with untreated sleep disorders, and 13.3% (33/245) felt that patients had only a limited understanding (Table 6).

**Table 6:** Patient awareness and understanding of sleep hygiene (N=245)

Question	Response options	Frequency (n)	Percentage (%)
How familiar do you think your patients are with sleep hygiene practices?	Not at all familiar	43	17.3%
	Somewhat familiar	95	38.1%
	Very familiar	107	42.9%
Do you think patients with sleep disorders understand the risks associated with untreated sleep disorders?	Yes	68	27.3%
	No	144	57.8%
	Somewhat	33	13.3%

Barriers to managing sleep disorders

Several barriers were identified in managing sleep disorders. The most common barrier was lack of patient awareness, cited by 32.9% (82/245) of respondents. Other challenges included limited time during consultations (27.8%, 69/245), resistance to lifestyle changes from patients (18.1%, 45/245), and limited access to mental health professionals (13.3%, 33/245). Limited access to diagnostic tools was reported by 15.3% (38/245) of practitioners (Table 7).

**Table 7:** Challenges in diagnosing and managing sleep disorders (N=245)

Question	Response options	Frequency (n)	Percentage (%)
What barriers do you face in managing sleep disorders in your patients?	Lack of patient awareness	82	32.9%
	Limited access to diagnostic tools	38	15.3%
	Limited time during consultations	69	27.8%
	Resistance to lifestyle changes from patients	45	18.1%
	Limited access to mental health professionals	33	13.3%

Closing questions

When asked whether sleep disorders were underdiagnosed and undertreated in their practice, 39.4% (98/245) of respondents agreed, while 45.3% (113/245) disagreed, and 13.6% (34/245) were unsure. In terms of further training, 36.9% (92/245) of participants expressed interest in additional training on sleep disorder management, while 63.1% (157/245) were not interested. Regarding the need for further research, 57.8% (144/245) agreed that more research on sleep disorders in family medicine settings was needed (Table 8).

**Table 8:** Perceptions on sleep disorder diagnosis, treatment, and research needs (N=245)

Question	Response options	Frequency (n)	Percentage (%)
Do you believe sleep disorders are underdiagnosed and undertreated in your practice?	Yes	98	39.4%
	No	113	45.3%
	Not sure	34	13.6%
Would you be interested in receiving further training on sleep disorder management?	Yes	92	36.9%
	No	157	63.1%
Do you think there is a need for more research on sleep disorders in family medicine settings?	Yes	144	57.8%
	No	47	18.9%
	Not sure	49	19.7%

## Discussion

This study sought to assess the prevalence and management of sleep disorders in family medicine practices in Saudi Arabia. Our results indicate that sleep disorders are prevalent among patients seen in primary care settings, but they are often underdiagnosed and undertreated. Despite the substantial impact of sleep disorders on health outcomes, family medicine practitioners face several challenges in effectively diagnosing and managing these conditions. These findings highlight the need for increased awareness, better training, and the implementation of standardized screening tools to improve the care of patients with sleep disorders.

The prevalence of sleep disorders observed in our study is consistent with existing research indicating that sleep-related problems are widespread among the general population [7-10]. In our cohort, 22.4% of family medicine practitioners reported experiencing frequent difficulty falling asleep or staying asleep, and 46.2% experienced excessive daytime sleepiness, which is consistent with the findings of other studies in Saudi Arabia, where sleep disturbances are reported in approximately 40% of the adult population. Similarly, a significant proportion of participants in our study (29%) reported symptoms such as snoring, gasping, or choking during sleep, which is in line with the high prevalence of obstructive sleep apnea in Saudi Arabia.

The reported prevalence of sleep disorders among practitioners aligns with global trends, where conditions like insomnia and sleep apnea are common [2-5]. This finding underscores the importance of family medicine practitioners being equipped to identify and manage these conditions, given that they are often the first point of contact for patients seeking help with sleep-related issues.

In our study, 44.2% of respondents reported using self-reported questionnaires, such as the PSQI or ESS, to screen for sleep disorders. While this is a promising practice, it is concerning that 28.1% of practitioners rely solely on clinical interviews, and only 11.3% use polysomnography or home sleep apnea testing (HSAT) for diagnosis. Polysomnography remains the gold standard for diagnosing sleep apnea, yet its high cost and limited availability in many primary care settings pose significant barriers. The reliance on self-reported questionnaires and clinical judgment, while useful, may lead to missed diagnoses, particularly for conditions like obstructive sleep apnea, which may not always be apparent through patient self-reporting [3-7].

These findings highlight the need for more widespread adoption of objective diagnostic tools, such as home sleep apnea testing, which is more accessible and cost-effective than polysomnography. Additionally, implementing routine screening for sleep disorders in primary care, particularly for high-risk groups, could help identify more patients who are currently undiagnosed [2-6].

Our study found that pharmacological treatment, particularly sedative medications and antidepressants, was commonly prescribed by family medicine practitioners (32.2% of respondents). This is consistent with previous studies indicating that pharmacological therapies, including benzodiazepines and zolpidem, are frequently used to treat sleep disorders in primary care settings. However, the overreliance on pharmacological treatments raises concerns about the long-term safety and potential for dependence. Lifestyle modifications, such as sleep hygiene and CBT-I, are considered first-line treatments for insomnia but were less frequently prescribed in our study (32.5% of respondents preferred lifestyle modifications).

The relatively low use of CBT-I and the underutilization of non-pharmacological interventions in our study mirror findings from other studies, which suggest that many primary care providers are either unaware of or lack access to these evidence-based treatments [10-13]; CBT-I has been shown to be highly effective in managing insomnia and should be more widely implemented, particularly in resource-limited settings. Educating family medicine practitioners about the efficacy and availability of CBT-I could reduce the reliance on medications and improve patient outcomes.

Our study identified several barriers to the effective management of sleep disorders, including lack of patient awareness, time constraints during consultations, resistance to lifestyle changes, and limited access to mental health professionals. These findings are consistent with the literature, which consistently highlights these barriers as significant challenges in primary care. A lack of patient awareness about sleep hygiene and the consequences of untreated sleep disorders was cited by 32.9% of respondents, while 27.8% reported time constraints during consultations as a major barrier.

These barriers point to the need for a multifaceted approach to improving sleep disorder management. Interventions should focus on increasing patient education, streamlining screening and diagnostic procedures to reduce time burden, and improving access to specialized care for conditions like sleep apnea. Additionally, training family medicine practitioners in the recognition of sleep disorders and the use of evidence-based treatments can help alleviate some of these challenges [14,15].

Family medicine practitioners are ideally positioned to address sleep disorders, as they manage patients with a wide range of medical conditions and have established relationships with them. However, our study suggests that many family medicine practitioners lack confidence in diagnosing and managing sleep disorders, likely due to inadequate training and exposure to sleep medicine during their medical education. This finding is consistent with other studies that have shown that primary care providers often feel ill-equipped to manage sleep disorders, despite their central role in managing chronic conditions that are closely related to sleep disturbances [8,10,12].

To address this gap, there is a need for enhanced education and training in sleep medicine for family medicine practitioners. Incorporating sleep medicine into medical curricula, offering continuing medical education (CME) courses on sleep disorders, and promoting collaboration between primary care providers and sleep specialists could improve the recognition and management of sleep disorders in primary care settings.

While this study provides valuable insights into the management of sleep disorders in family medicine practices, it is not without limitations. The study relied on self-reported data, which may be subject to recall bias or social desirability bias. Additionally, the study was conducted in a specific region of Saudi Arabia, and the findings may not be generalizable to other settings or countries. Future studies with larger, more diverse samples are needed to validate these findings and explore the broader applicability of the results.

## Conclusions

In conclusion, this study highlights the significant prevalence of sleep disorders in family medicine practices in Saudi Arabia and the challenges family medicine practitioners face in diagnosing and managing these conditions effectively. Despite the availability of screening tools and treatment options, sleep disorders remain underdiagnosed and undertreated due to barriers such as limited time, patient awareness, and access to specialized care. Enhancing practitioner education, expanding the use of non-pharmacological treatments like CBT for insomnia, and integrating routine sleep disorder screening into primary care are crucial steps to improving patient outcomes. Future research should focus on developing practical solutions to overcome these barriers and evaluate the impact of targeted interventions on both practitioners and patients.

## References

[1] Alhejaili F, Kanbr O, Jastaniah N (2024). Sleep disorders among elderly in Saudi Arabia: a cross-sectional study. Ann Thorac Med.

[2] Alomri RM, Alghamdi Y (2024). The prevalence and predictors of sleep disorders and their impact on academic performance among Saudi university students: a cross-sectional study. Cureus.

[3] McArdle N, Reynolds AC, Hillman D, Moses E, Maddison K, Melton P, Eastwood P (2022). Prevalence of common sleep disorders in a middle-aged community sample. J Clin Sleep Med.

[4] Hakami A, Hakami RA, Al-Amer MA, Sharahili LM, Zuqayl AH, Hakami TK, Dighriri IM (2023). Prevalence of sleep disorders among the general population of the Jazan region of Southwest Saudi Arabia. Cureus.

[5] Syed W, Al-Rawi MB (2023). Assessment of sleeping disorders, characteristics, and sleeping medication use among pharmacy students in Saudi Arabia: a cross-sectional quantitative study. Med Sci Monit.

[6] AlAteeq MA, Alghaihab MM, Marghlani LK, Shamsaddin LA, Alghamdi RK, Alfadley MA (2024). Comparing the prevalence of sleep disorders among underweight, normal, overweight, and obese adults in Riyadh, Saudi Arabia. Cureus.

[7] Alkahtani RF, Alomar AA, Alkanhal AF, Alhinti MF, Alatoui SE, Alrashidi RR, Saleh A (2022). Prevalence of anxiety, depression, and sleep disturbances associated with the COVID-19 outbreak in Riyadh, Saudi Arabia. Cureus.

[8] Almajid H, Elnasieh AM, Alnamlah AA (2024). Prevalence and associated risk factors of insomnia among adults in Riyadh, Saudi Arabia. Cureus.

[9] Abdelmoaty Goweda R, Hassan-Hussein A, Ali Alqahtani M (2020). Prevalence of sleep disorders among medical students of Umm Al-Qura University, Makkah, Kingdom of Saudi Arabia. J Public Health Res.

[10] Alkalash SH, Aldawsari AK, Alfahmi SS (2023). The prevalence of nomophobia and its impact on academic performance of medical undergraduates at the College of Medicine, Umm Al-Qura University, Makkah City, Saudi Arabia. Cureus.

[11] Kabel AM, Al Thumali AM, Aldowiala KA, Habib RD, Aljuaid SS (2018). Sleep disorders in a sample of students in Taif University, Saudi Arabia: the role of obesity, insulin resistance, anemia and high altitude. Diabetes Metab Syndr.

[12] AlEidan A, Al-Shamrani M, AlGhofaily M (2023). Prevalence of sleep problems and habits among children in Saudi Arabia: a cross-sectional study. Saudi Med J.

[13] Pirzada A, Awadh AA, Aleissi SA, Almeneessier AS, BaHammam AS (2020). Reopening sleep medicine services in the conundrum of an ongoing COVID-19 pandemic: a global view. Sleep Vigil.

[14] Al-Qahtani FS (2024). Prevalence of sleep disturbance and its associated factors among diabetes type-2 patients in Saudi Arabia. Front Public Health.

[15] Saleem AH, Al Rashed FA, Alkharboush GA, Almazyed OM, Olaish AH, Almeneessier AS, BaHammam AS (2017). Primary care physicians' knowledge of sleep medicine and barriers to transfer of patients with sleep disorders. A cross-sectional study. Saudi Med J.

